# Species diversity of *Ganoderma* (Ganodermataceae, Polyporales) with three new species and a key to *Ganoderma* in Yunnan Province, China

**DOI:** 10.3389/fmicb.2022.1035434

**Published:** 2022-10-14

**Authors:** Jun He, Xiao Han, Zong-Long Luo, E-Xian Li, Song-Ming Tang, Hong-Mei Luo, Kai-Yang Niu, Xi-jun Su, Shu-Hong Li

**Affiliations:** ^1^Yunnan Academy of Agricultural Sciences, Biotechnology and Germplasm Resources Institute, Kunming, China; ^2^College of Agronomy and Biosciences, Dali University, Dali, Yunnan, China

**Keywords:** 3 new taxa, basidiomycetes, Lingzhi, medicinal mushroom, multigene phylogeny, taxonomy

## Abstract

*Ganoderma* is a globally distributed genus that encompasses species with forestry ecological, medicinal, economic, and cultural importance. Despite the importance of this fungus, the studies on the species diversity of *Ganoderma* in Yunnan Province, China (YPC) have poorly been carried out. During this study, opportunistic sampling was used to collect 21 specimens of *Ganoderma* from YPC. Morphology and multigene phylogeny of the internal transcribed spacer (ITS) regions, the large subunit of nuclear ribosomal RNA gene (nrLSU), the translation elongation factor 1-α gene (TEF1-α), and the second largest subunit of RNA polymerase II (RPB2) were used to identify them. Morphological and molecular characterization of the 21 specimens showed that they belong to 18 species of *Ganoderma*, of which three are novel *viz. G. artocarpicola*, *G. obscuratum* and *G. yunnanense*. *Ganoderma artocarpicola* is characterized by the sessile and concrescent basidiomata, reddish brown to yellowish brown pileus surface, heterogeneous context, wavy margin, and ovoid basidiospores. *Ganoderma obscuratum* is distinguished by small pores (6–9 per mm), dorsolaterally sub-stipitate basidiomata which become greyish-brown when dry, and narrow ellipsoid basidiospores. *Ganoderma yunnanense* is characterized by cream color pore surface and context, centrally to laterally stipitate basidiomata with reddish-brown to violet-brown strongly laccate pileus surface, and broadly ellipsoid basidiospores. With the help of an extensive literature survey and the results of this study, a checklist of 32 *Ganoderma* species from YPC was established, which accounts for 71.11% of the known species in China. In addition, a key to the *Ganoderma* in YPC is also provided.

## Introduction

*Ganoderma* P. Karst. 1881 is a genus of white rot fungi in the Polyporales and Ganodermataceae containing species that were originally described in the United Kingdom (Moncalvo and Ryvarden, 1997). *Ganoderma* worldwide distribution from warm temperate to tropical, and is a facultative parasite on living, dead or rotting trees (Zhou et al., 2015). *Ganoderma* species cause white rot of hardwoods by decomposing lignin, cellulose, and related polysaccharides. Generally associated with the decay of roots and the lower trunk or stems flare, which can lead to hazardous tree conditions and tree failures, resulting in serious damage to property and life (Loyd et al., 2017). Previous studies have reported that some species of *Ganoderma* can cause diseases as pathogens of living trees such as *Areca catechu* (betel nut palm), *Elaeis guineensis* (oil palm), *Hevea brasiliensis* (rubber), and cause wood rot of forest trees and can contribute to tree mortality and failure by wind throw (Adaskaveg et al., 1991; Elliott and Broschat, 2001; Tonjock and Afui, 2015). Several species are responsible for stem and butt rots of commercially important crops such as stem rot of betel nut palm and oil palm caused by *G. boninense* or *G. zonatum* (Elliott and Broschat, 2001; Nur et al., 2019), and rubber root rot caused by *G. philippi* (Glen et al., 2009). Other species, such as *G. australe*, *G. sessile* and *G. curtisii*, seem to be opportunistic pathogens and typically only cause serious decay in old or stressed trees (Sinclair and Lyon, 2005). On the other hand, some of *Ganoderma* have been shown to selectively delignify wood and are recognized as a potentially important source of lignin degrading enzymes (Otjen et al., 1987). Obviously, *Ganoderma* are ecologically indispensable, but some of them are pathogenic and can cause diseases in forest trees.

Moreover, most *Ganoderma* species have biologically active components with nutritional and medicinal effects, which are economically important (Dai et al., 2009). *Ganoderma* has been used in Asian countries for over two millennia as a traditional medicine for maintaining vivacity and longevity, for its perceived health benefits, has gained wide popular use as a dietary supplement (Hapuarachchi et al., 2018a). *Ganoderma lucidum* (“lingzhi”) and *G. sinense* have been included in the Chinese Pharmacopoeia, and are used for anti-cancer treatment, lowering blood pressure, and improving immunity (Dai et al., 2009; Sun et al., 2022). Research of *Ganoderma* is a hot topic since its high potential to use in biotechnology.

As a consequence of several taxonomic and molecular phylogenetic studies on *Ganoderma*, an unexpectedly high level of species diversity has been uncovered worldwide, with the description of many new species (Cao et al., 2012; Cao and Yuan, 2013; Li et al., 2015; Xing et al., 2016, 2018; Hapuarachchi et al., 2018b, 2019; Liu et al., 2019; Wu et al., 2020; He et al., 2021). However, many taxonomy confusions have resulted from the great variability in the macroscopic characters of the *Ganoderma* basidiomata. As of 20 September 2022, there were 488 records of *Ganoderma* recorded in Index Fungorum,^1^ and 529 records in MycoBank.^2^ Nearly two-thirds of these records have been identified as synonyms. Up to now, 181 species are taxonomically accepted in *Ganoderma*, making it as one of the most species-rich genera in Ganodermataceae (Costa-Rezende et al., 2020). The genus is unique with characteristic double-walled basidiospores with a thin hyaline exosporium and ornamented endospore (Karsten, 1881; Moncalvo and Ryvarden, 1997).

**Figure 1:**
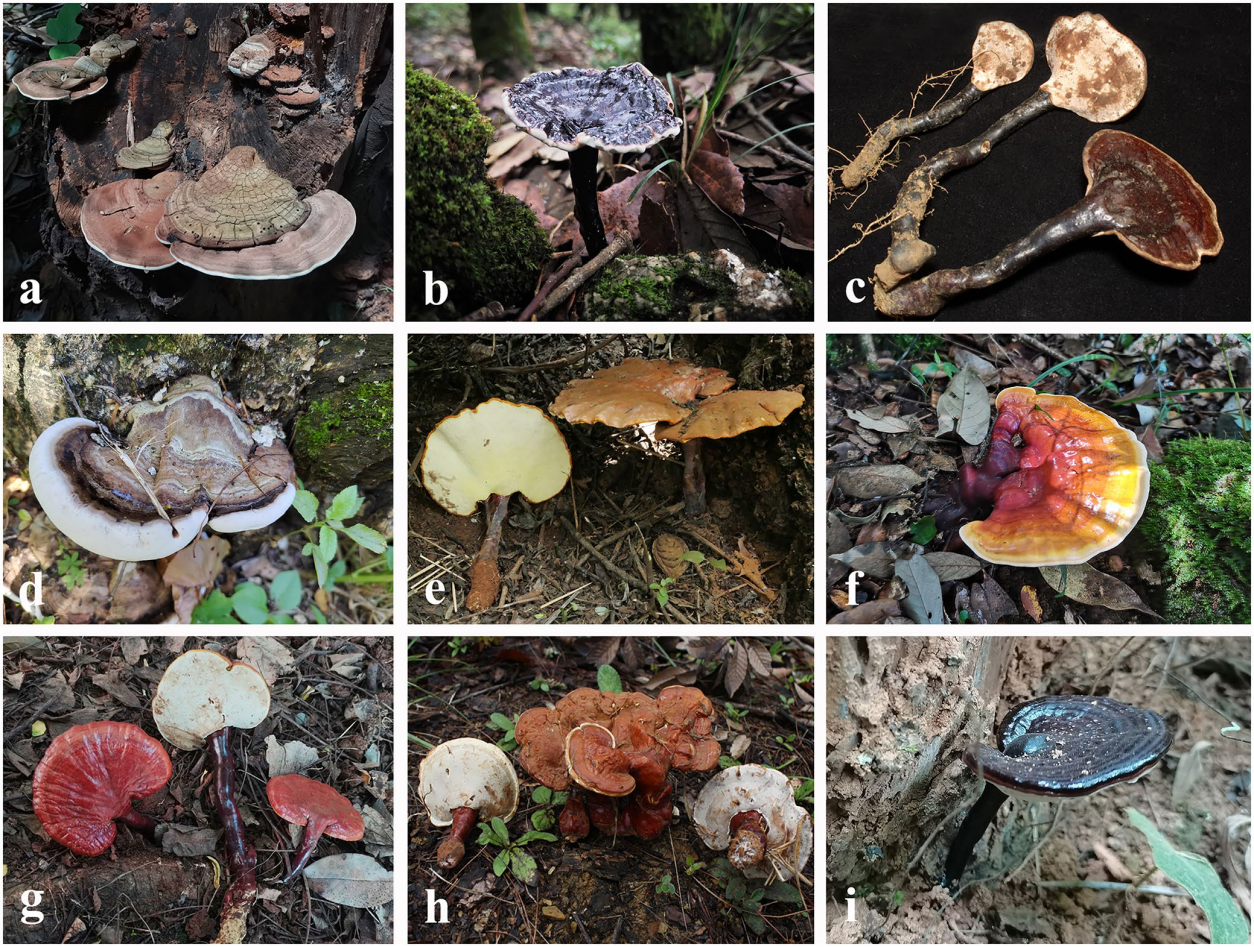
Basidiomata of the *Ganoderma* species collected in Yunnan Province, China. **(A)** *Ganoderma applanatum* found in *Eriobotrya* tree (HKAS123785); **(B)** *Ganoderma dianzhongense* in *Cyclobalanopsis* tree (HKAS 112719); **(C)** *Ganoderma esculentum* (HKAS123789); **(D)** *Ganoderma gibbosum* in *Carya* tree (HKAS123781); **(E)** *Ganoderma lingzhi* in *Prunus* tree (HKAS123768); **(F)** *Ganoderma leucocontextum* in *Cyclobalanopsis* tree (HKAS123767); **(G)** *Ganoderma lucidum* in *Quercus* tree (HKAS123773); **(H)** *Ganoderma multipileum* (HKAS123775); **(I)** *Ganoderma sinense* in *Acer* tree (HKAS123770). Photographs were taken by JH.

China has a complex and diverse plant diversity, and a diversified three-dimensional climate environment that breeds abundant wild *Ganoderma* resources, thus, a total of 40 species of *Ganoderma* have been reported in China (Cao et al., 2012; Cao and Yuan, 2013; Li et al., 2015; Xing et al., 2018; Hapuarachchi et al., 2018b, 2019; Liu et al., 2019; Wu et al., 2020; He et al., 2021; Sun et al., 2022). Yunnan is an inland Province with low latitude and high altitudes in southwest China, which is a hotspot of global biodiversity and has abundant wildlife resources Nine type species of *Ganoderma viz. Ganoderma alpinum*, *G. chuxiongense*, *G. dianzhongense*, *G. esculentum*, *G. mutabile*, *G. puerense*, *G. subangustisporum*, *G. weixiense* and *G. yunlingense* have been reported in this region. In addition, several researchers have reported the diversity of *Ganoderma* in southwestern China, such as Luangharn et al. (2021), which reported 13 *Ganoderm*a species *viz. G. applanatum*, *G. australe*, *G. calidophilum*, *G. flexipes*, *G. gibbosum*, *G. leucocontextum*, *G. lucidum*, *G. multiplicatum*, *G. resinaceum*, *G. sanduense*, *G. sichuanense*, *G. sinense*, and *G. tsugae* from YPC based on comprehensive morphological characteristics and molecular analyses. Apparently, there are many economically and medicinally important *Ganoderma* species in YPC (Figure 1; He et al., 2021; Luangharn et al., 2021; Sun et al., 2022). However, with the exception of the taxonomic and new species description studies, very little efforts have been made to identify the *Ganoderma* species diversity in YPC. Thus, the objectives of this research are, to identify and describe different species of *Ganoderma* including three new species in YPC based on morphology and multigene phylogeny, and to prepare a checklist of *Ganoderma* and a key to *Ganoderma* in YPC.

## Materials and methods

### Specimen collection

Twenty-one *Ganoderma* specimens were collected during the rainy season from July 2016 to September 2021 from jungle hill forests in Yunnan Province, China. They were photographed in the field, then collected and wrapped in aluminium foils or kept separately in a plastic collection box. Macro-morphology of fresh basidiomata was described, on the same day of collection. Specimens were then thoroughly dried at 40°C in a food drier, stored in sealed plastic bags with anhydrous silica gel, and deposited in the herbarium of Kunming Institute of Botany, Chinese Academy of Sciences Academia Sinica (HKAS section, KUN). MycoBank numbers were obtained as described in Jayasiri et al. (2015).

### Morphological study

Macro-morphological studies were conducted following the protocols provided by Torres-Torres and Guzmán-Dávalos (2012). Key colors were obtained from Kornerup and Wanscher (1978). Micro-morphological data were obtained from the dried specimens and observed under a light microscope (Nikon). The temporary prepared microscope slides were placed under magnification up to 1,000 × using Nikon ECLIPSE80i (Nikon, Japan) compound stereomicroscope for observation and microscopic morphological photography. Microscopic observations were made from slide preparations stained with 10% potassium hydroxide (KOH), Melzer’s reagent, and Cotton Blue. Measurements were made using the Image Frame work v.0.9.7 To represent variation in the size of basidiospores, 5% of measurements were excluded from each end of the range and extreme values were given in parentheses (He et al., 2021).

The following abbreviations are used: IKI = Melzer’s reagent, IKI– = neither amyloid nor dextrinoid, KOH = 10% potassium hydroxide, CB = Cotton Blue, CB + = cyanophilous, L = mean spore length (arithmetic average of all spores), W = mean spore width (arithmetic average of all spores). The abbreviation for spore measurements (*x*/*y*/*z*) denote “x” spores measured from “y” basidiocarps of “z” specimens. Basidiospore dimensions (and “*Q*” values) are given as (a) b–*av*–c (d). Where “a” and “d” refer to the lower and upper extremes of all measurements, respectively, b-c the range of 95% of the measured values, and *Q* is the length/width ratio of basidiospores, is given as *Q*_m_ ± standard deviation, where *Q*_m_ is the average *Q* of all basidiospores. Where “a” and “d” refer to the lower and upper extremes of all measurements, “*av*” is the average “b,” respectively, b-c are the range of 95% of the measured values, and *Q* is the length/width ratio of basidiospores, which is given as *Q*_m_ ± standard deviation, where *Q*_m_ is the average *Q* of all basidiospores.

### DNA extraction, PCR amplification, and sequencing

Genomic DNA isolation and PCR of the studied material were performed at the Yunnan Academy of Agricultural Sciences, China. Genomic DNA was extracted from dried specimens using Ezup Column Fungi Genomic DNA Purification Kit (Sangon Biotech Limited Company, Kunming, Yunnan, China) based on the manufacturer’s protocol. Primer pairs used for PCR were ITS1F/ITS5 (White et al., 1990) for ITS, LR5/LR0R (Vilgalys and Hester, 1990) for nrLSU, TEF1–983/TEF1–1567R (Matheny et al., 2007) for TEF1–α, and RPB2–6f/fRPB2–7cR (Liu et al., 1999) for RPB2. Primer sequences of the primers used in this study are available in the WASABI database of the AFTOL website (aftol.org). Gene regions were amplified in 30 μl reactions containing 15 μl 2 × Taq Plus Master Mix II (Sangon Biotechnology Co., Kunming, China), 13 μl ddH2O, 0.5 μl 10 μM of forward and reverse primers, 1 μl DNA. PCR conditions were used as in the Table 1, using a C1000 thermal cycler (Bio-Rad China). The PCR amplicons were sent to Sangon Biotech (China) for Sanger sequencing. Raw DNA sequences were assembled, and edited in Sequencher 4.1.4 and the assembled DNA sequences were deposited in GenBank (Table 2).

**Table 1:** PCR primers and conditions used in this study.

Locus	Primers	PCR conditions^#^	References
ITS	ITS1F, ITS4	94°C: 30 s, 53°C: 30 s, 72°C: 50 s. (38 cycles)	White et al. (1990)
nrLSU	LR0R, LR5	94°C: 30 s, 52°C: 30 s, 72°C: 1 min. (38 cycles)	Vilgalys and Hester (1990)
TEF1–α	983F, 1567R	94°C: 30 s, 52°C: 1 min, 72°C: 1 min. (38 cycles)	Matheny et al. (2007)
RPB2	RPB2-6F, f RPB2-7cR	94°C: 30 s, 58°C: 30 s, 72°C: 1 min. (38 cycles)	Liu et al. (1999)

# The three steps given for each primer pair were repeated for 38 cycles, preceded by an initial denaturation step of 5 min at 94°C, and followed by a final elongation step of 10 min at 72°C and a final hold at 4°C.

**Table 2:** Specimens used for phylogenetic analyses and their corresponding GenBank accession numbers.

Species	Voucher/strain	Origin	GenBank accession numbers			
			ITS	nLSU	TEF1–α	RPB2
*Ganoderma acaciicola*	Cui 16,815 ^T^	Australia	MZ354895	MZ355005	–	MZ245384
*G. acaciicola*	Cui 16,813	Australia	MZ354893	MZ355003	–	MZ245382
*G. acontextum*	JV 0611/21G ^T^	Guatemala	KF605667	–	MG367538	MG367489
*G. acontextum*	JV 1208/11 J	Guatemala	KF605668	–	MG367540	MG367490
*G. adspersum*	HSBU-200894	China	MG279154	–	MG367542	–
*G. adspersum*	Dai 13,191	China	MG279153	–	MG367541	MG367492
*G. alpinum*	Cui 17,467 ^T^	Yunnan, China	MZ354912	–	–	–
*G. alpinum*	Cui 18,402	Yunnan, China	MZ354910	–	–	–
*G. angustisporum*	Cui 13,817 ^T^	Fujian, China	MG279170	MZ355090	MG367563	MG367507
*G. angustisporum*	Cui 18,240	Malaysia	MZ354979	MZ355074	MZ221634	MZ245386
***G. applanatum***	**L5370**	**Yunnan, China**	**ON994241**	**OP380254**	**OP508448**	**–**
*G. applanatum*	SFC20150930-02	Inje gun,Gangwon do	KY364258	–	KY393288	KY393274
***G. artocarpicola***	**HL173** ^**T**^	**Yunnan, China**	ON994239*	OP456495*	OP508442*	OP508428*
***G. artocarpicola***	**HL188**	**Yunnan, China**	ON994240*	OP380253*	OP508441*	OP508427*
*G. aridicola*	Dai 12,588 ^T^	South Africa	KU572491	–	KU572502	–
*G. australe*	DHCR417 HUEFS	Australia	MF436676	MF436673	MF436678	–
*G. australe*	DHCR411 HUEFS	Australia	MF436675	MF436672	MF436677	–
*G. austroafricanum*	CBS138724 ^T^	South Africa	KM507324	KM507325	–	MK611970
*G. austroafricanum*	CMW25884	South Africa	MH571693	–	MH567296	–
*G. bambusicola*	Wu 1,207–152	Taiwan, China	MN957782	–	LC517942	LC517945
*G. bambusicola*	Wu 1,207–151	Taiwan, China	MN957781	–	LC517941	LC517944
*G. boninense*	WD 2085	Japan	KJ143906	–	KJ143925	KJ143965
*G. boninense*	WD 2028	Japan	KJ143905	KU220015	KJ143924	KJ143964
*G. brownii*	JV 1105/9 J	United States	MG279159	–	MG367547	MG367494
*G. brownii*	JV 0709/109	United States	KF605662	–	MG367548	MG367495
*G. bubalinomarginatum*	Dai 20,075 ^T^	Guangxi, China	MZ354926	MZ355010	MZ221637	MZ245388
*G. bubalinomarginatum*	Dai 20,074	Guangxi, China	MZ354927	MZ355040	MZ221638	MZ245389
*G. calidophilum*	MFLU 19–2,174	Yunnan, China	MN398337	–	–	–
***G. calidophilum***	**H36**	**Yunnan, China**	**MW750241**	**OP380255**	**MW838997**	**MW839003**
*G. carnosum*	JV 8709 8	Czech R, Europe	KU572493	–	–	–
*G. carnosum*	MJ 21 08	Czech R, Europe	KU572492	–	–	–
*G. carocalcareum*	DMC 513	Cameroon	EU089970	–	–	–
*G. carocalcareum*	DMC 322 ^T^	Cameroon	EU089969	–	–	–
*G. casuarinicola*	HKAS 104639	Thailand	MK817650	MK817654	MK871328	MK840868
*G. casuarinicola*	Dai 16,336 ^T^	Guangdong, China	MG279173	–	MG367565	MG367508
*G. chocoense*	QCAM3123 ^T^	Ecuador	MH890527	–	–	–
*G. chuxiongense*	Cui 17,262 ^T^	MZ354907	MZ354907	–	–	–
*G. cocoicola*	Cui 16,791 ^T^	Australia	MZ354984	MZ355091	MZ221643	MZ245393
*G. cocoicola*	Cui 16,792	Australia	MZ354985	MZ355092	MZ221644	MZ245394
*G. concinnum*	Robledo 3,235	Brazil	MN077523	MN077557	–	–
*G. concinnum*	Robledo 3,192	Brazil	MN077522	MN077556	–	–
*G. curtisii*	CBS 100132	NC, United States	JQ781849	–	KJ143927	KJ143967
*G. curtisii*	CBS 100131	NC, United States	JQ781848	–	KJ143926	KJ143966
*G. destructans*	CBS 139793 ^T^	South Africa	NR132919	NG058157	–	–
*G. destructans*	Dai 16,431	South Africa	MG279177	–	MG367569	MG367512
***G. dianzhongense***	**L4331** ^T^	**Yunnan, China**	**MW750237**	**OP380256**	**MW838993**	**MZ467043**
*G. dianzhongense*	L4969	Yunnan, China	MW750240	–	MW838996	MZ467044
*G. dianzhongense*	L4759	Yunnan_China	MW750239	–	MW838995	MW839001
*G. dunense*	CMW 42150	South Africa	MG020249	–	MG020228	–
*G. dunense*	CMW 42157 ^T^	South Africa	MG020255	–	MG020227	–
*G. ecuadorense*	URM 89449	Ecuador	MK119828	MK119908	MK121577	MK121535
*G. ecuadorense*	URM 89441	Ecuador	MK119827	MK119907	MK121576	MK121534
*G. eickeri*	CMW 49692 ^T^	South Africa	MH571690	–	MH567287	–
*G. eickeri*	CMW 50325	South Africa	MH571689	–	MH567290	–
*G. ellipsoideum*	GACP1408966 ^T^	Hainan, China	MH106867	–	–	–
*G. ellipsoideum*	Dai 20,544	China	MZ354971	MZ355033	MZ221654	MZ245400
***G. ellipsoideum***	**L4954**	**Yunnan, China**	**ON994242**	**OP380257**	**OP508446**	**–**
*G. enigmaticum*	Dai 15,971	Africa	KU572487	–	KU572497	MG367514
*G. enigmaticum*	Dai 15,970	Africa	KU572486	–	KU572496	MG367513
*G. esculentum*	L4935 ^T^	Yunnan, China	MW750242	–	MW838998	MW839004
***G. esculentum***	**HL107**	**Yunnan, China**	**ON994243**	**OP380258**	**OP508437**	**OP508424**
*G. fallax*	JV 1009/27 ^T^	United States	KF605655	–	–	–
*G. fallax*	JV 0709/39	United States	KF605658	–	–	–
*G. flexipes*	Cui 13,841	Hainan, China	MZ354923	MZ355063	MZ221655	MZ245401
***G. flexipes***	**HL137**	**Yunnan, China**	**ON994244**	**OP380259**	**OP508439**	**OP508426**
*G. fornicatum*	BCRC35374	Taiwan	JX840349	**–**	**–**	**–**
*G. gibbosum*	Cui 13,940	China	MZ354972	MZ355021	MZ221658	MZ245404
***G. gibbosum***	**HL10**	**Yunnan, China**	**ON994245**	**OP380260**	**OP508434**	**OP508421**
*G. guangxiense*	Cui 14,453 T	Guangxi, China	MZ354939	MZ355037	MZ221661	MZ245407
*G. guangxiense*	Cui 14,454	Guangxi, China	MZ354941	MZ355039	MZ221662	MZ245408
*G. heohnelianum*	Cui 13,982	Guangxi, China	MG279178	–	MG367570	MG367515
*G. heohnelianum*	Dai 11,995	Yunnan, China	KU219988	KU220016	MG367550	MG367497
*G. hochiminhense*	MFLU 19–2,225	Vietnam	MN396662	MN396391	MN423177	–
*G. hochiminhense*	MFLU 19–2,224 ^T^	Vietnam	MN398324	MN396390	MN423176	–
*G. knysnamense*	CMW 47756	South Africa	MH571684	–	MH567274	–
*G. knysnamense*	CMW 47755 ^T^	South Africa	MH571681	–	MH567261	–
*G. leucocontextum*	GDGM 40200	China	KF011548	–	–	–
***G. leucocontextum***	**L4913**	**Yunnan, China**	**ON994246**	**OP380261**	**OP508445**	**OP508431**
*G. lingzhi*	Dai 20,895	Liaoning, China	MZ354904	MZ355006	MZ221668	MZ245413
***G. lingzhi***	**HL56**	**Yunnan, China**	**ON994247**	**OP380262**	**–**	**OP508423**
*G. lobatum*	JV 1008 32	United States	KF605670	–	MG367554	MG367500
*G. lobatum*	JV 1008 31	United States	KF605671	–	MG367553	MG367499
*G. lucidum*	Cui 14,404	Sichuan, China	MG279181	MZ355051	MG367573	MG367519
***G. lucidum***	**L5478**	**Yunnan, China**	**ON994248**	**OP380263**	**OP508449**	**OP508433**
*G. magniporum*	Zhou 439	Guangxi, China	MZ354936	MZ355097	–	–
*G. magniporum*	Dai 19,966	Yunnan, China	–	MZ355098	MZ221670	MZ345728
*G. martinicense*	246TX	TX, United States	MG654185	–	MG754737	MG754858
*G. martinicense*	LIP SW-Mart08-55 ^T^	Martinique, France	KF963256	–	–	–
*G. mastoporum*	TNM-F0018838	China	JX840350	–	–	–
*G. mexicanum*	MUCL 55832	Martinique	MK531815	–	MK531829	MK531839
*G. mexicanum*	MUCL 49453	Martinique	MK531811	–	MK531825	MK531836
*G. mirabile*	Cui 18,271	Malaysia	MZ354958	MZ355067	MZ221672	MZ345729
*G. mirabile*	Cui 18,283	Malaysia	MZ354959	MZ355069	MZ221673	MZ345730
*G. mizoramense*	UMN MZ5	India	KY643751	KY747490	–	–
*G. mizoramense*	UMN MZ4 ^T^	India	KY643750	–	–	–
*G. multipileum*	Cui 13,597	Hainan, China	MZ354899	MZ355043	MZ221675	MZ345732
***G. multipileum***	**L4989**	**Yunnan, China**	**ON994249**	**OP380264**	**OP508447**	**OP508432**
*G. multiplicatum*	CC8	China	KU569515	KU570915	–	–
*G. multiplicatum*	Dai 17,395	Brazil	MZ354903	–	MZ221678	MZ345734
*G. mutabile*	Yuan 2,289 ^T^	Yunnan, China	JN383977	–	–	–
*G. mutabile*	Dai 20,414	China	MZ354977	MZ355110	MZ221680	MZ345735
*G. myanmarense*	MFLU 19–2,167 ^T^	Myanmar	MN396330	MN428672	–	–
*G. myanmarense*	MFLU 19–2,169	Myanmar	MN396329	MN398325	–	–
*G. nasalanense*	GACP17060211 ^T^	Laos	MK345441	MK346831	–	–
*G. nasalanense*	GACP17060212	Laos	MK345442	MK346832	–	–
*G. neojaponicum*	FFPRI WD 1532	Chiba, Japan	MN957785	–	–	–
*G. neojaponicum*	FFPRI WD 1285	Tokyo, Japan	MN957784	–	–	–
***G. obscuratum***	**Lsh88** ^**T**^	**Yunnan, China**	ON994237*	OP456493*	OP508450*	–
***G. obscuratum***	**Lsh89**	**Yunnan, China**	ON994238*	OP456494*	OP508451*	–
*G. orbiforme*	Cui 13,918	Hainan, China	MG279186	–	MG367576	MG367522
***G. orbiforme***	**HL43**	**Yunnan, China**	**ON994250**	**OP380265**	**OP508435**	**–**
*G. oregonense*	CBS 266.88	United States	JQ781876	–	–	KJ143975
*G. oregonense*	CBS 265.88	United States	JQ781875	–	KJ143933	KJ143974
*G. ovisporum*	HKAS 123193 ^T^	China	MZ519547	MZ519545	**–**	MZ547661
*G. ovisporum*	GACP 20071602	China	MZ519548	MZ519546	–	MZ547662
*G. parvulum*	MUCL 52655	Guiana, French	MK554770	–	MK554717	MK554755
*G. parvulum*	MUCL 47096	Cuba	MK554783	–	MK554721	MK554742
*G. pfeifferi*	JV 0511/11	United States	KF605660	–	–	–
*G. pfeifferi*	120,818	British	AY884185	–	–	–
*G. philippii*	Cui 14,443	Hainan, China	MG279188	–	MG367578	MG367524
*G. philippii*	MFLU 19–2,222	Thailand	MN401410	MN398326	MN423174	–
*G. podocarpense*	QCAM 6422 ^T^	Panama	MF796661	–	–	–
*G. podocarpense*	JV 1504/126	Costa Rica	MZ354942	–	MZ221687	MZ345737
*G. polychromum*	330OR	OR, United States	MG654196	–	MG754742	–
*G. polychromum*	MS343OR	OR, United States	MG654197	–	MG754743	–
*G. puerense*	Dai 20,427 ^T^	Yunnan, China	–	MZ355012	MZ221688	MZ345738
*G. ravenelii*	MS187FL	FL, United States	MG654211	–	MG754745	MG754865
*G. ravenelii*	NC-8349	United States	AY456341	–	–	–
*G. resinaceum*	LGAM 462	Greece	MG706250	MG706196	MG837858	MG837821
*G. resinaceum*	LGAM 448	Greece	MG706249	MG706195	MG837857	MG837820
*G. ryvardenii*	HKAS 58053 ^T^	South Africa	HM138670	–	–	–
*G. ryvardenii*	HKAS 58054	South Africa	HM138671	–	–	–
*G. sandunense*	GACP 18012501 ^T^	China	MK345450	–	–	–
***G. sandunense***	**L4906**	**Yunnan, China**	**ON994251**	**OP380266**	**OP508444**	**OP508430**
*G. sessile*	113FL	FL, United States	MG654307	–	MG754748	MG754867
*G. sessile*	111TX	TX, United States	MG654306	–	MG754747	MG754866
*G. shanxiense*	BJTC FM423 ^T^	Shangxi, China	MK764268	–	MK783937	MK783940
*G. shanxiense*	Dai 18,921	Shangxi, China	MZ354909	MZ355044	MZ221691	MZ345740
*G. sichuanense*	Cui 16,343	China	MZ354928	MZ355011	MZ221692	MZ345741
*G. sichuanense*	Dai 19,651	Sri Lanka	MZ354929	MZ355031	MZ221693	MZ345742
*G. sinense*	Wei 5,327	Hainan, China	KF494998	KF495008	KF494976	MG367529
***G. sinense***	**HL109**	**Yunnan, China**	**ON994252**	**OP380267**	**OP508438**	**OP508425**
*G. steyaertanum*	MEL 2382783	Australia	KP012964	–	–	–
*G. steyaertanum*	6 WN-20B	Indonesia	KJ654462	–	–	–
*G. subangustisporum*	Cui 18,592 ^T^	Yunnan, China	MZ354981	MZ355027	MZ221697	–
*G. subangustisporum*	Cui 18,597	Yunnan, China	MZ354980	MZ355025	MZ221700	MZ345746
*G. thailandicum*	HKAS 104640 ^T^	Thailand	MK848681	MK849879	MK875829	MK875831
*G. thailandicum*	HKAS 104641	Thailand	MK848682	MK849880	MK875830	MK875832
*G. tongshanense*	Cui 17,168 ^T^	Hubei, China	MZ354975	MZ355024	MZ221706	–
*G. tornatum*	TBG01AM2009	Brazil	JQ514108	JX310808	–	–
*G. tornatum*	URM 82776	Brazil	JQ514110	JX310800	–	–
*G. tropicum*	Dai 16,434	Hainan, China	MG279194	MZ355026	MG367585	MG367532
*G. tropicum*	Dai 19,679	China	MZ354900	MZ355009	MZ221707	MZ358825
***G. tropicum***	**HL186**	**Yunnan, China**	**ON994253**	**OP380268**	**OP508440**	–
*G. tsugae*	Dai 12,760	CT, United States	KJ143920	–	KJ143940	KJ143978
*G. tsugae*	HKAS 97406	Yunnan, China	MG279195	–	MG367586	MG367533
*G. tuberculosum*	GVL 40	Veracruz, Mexico	MT232634	–	–	–
*G. tuberculosum*	JV 1607/62	Costa Rica	MZ354944	MZ355087	MZ221710	–
*G. weberianum*	CBS 219 36	Philippines	MK603804	–	MK611974	MK611972
*G. weberianum*	CBS 128581	Taiwan, China	MK603805	–	MK636693	MK611971
*G. weberianum*	Dai 19,673	China	MZ354930	MZ355032	MZ221712	MZ358829
*G. weberianum*	Dai 19,682	China	MZ354932	MZ355042	MZ221713	MZ358830
*G. weixiense*	HKAS 100649 ^T^	Yunnan, China	MK302444	MK302446	MK302442	–
*G. weixiense*	HKAS 100650	Yunnan, China	MK302445	MK302447	MK302443	–
*G. wiiroense*	UMN 21 GHA ^T^	Ghana	KT952363	KT952364	–	–
*G. wiiroense*	UMN 20 GHA	Ghana	KT952361	KT952362	–	–
*G. yunlingense*	Cui 16,288 ^T^	Yunnan, China	MZ354915	MZ355077	MZ221718	–
*G. yunlingense*	Cui 17,043	Yunnan, China	MZ354916	MZ355078	MZ221719	–
***G. yunnanense***	**HL45** ^**T**^	**Yunnan, China**	**ON994235** ^*^	**OP373192** ^*^	**OP508436** ^*^	**OP508422** ^*^
***G. yunnanense***	**L4812**	**Yunnan, China**	**ON994236** ^*^	**OP373193** ^*^	**OP508443** ^*^	**OP508429** ^*^
*G. zonatum*	FL 03	FL, United States	KJ143922	–	KJ143942	KJ143980
*G. zonatum*	FL 02	FL, United States	KJ143921	–	KJ143941	KJ143979
*Amauroderma rugosum*	Cui 9,011	Guangdong, China	KJ531664	–	KU572504	MG367506
*Sanguinoderma rude*	Cui 16,592	Australia	MK119836	MK119916	MK121586	MK121521

The newly generated sequences are shown in black bold. Superscript “T” is used after the number to show the type specimens. ^*^New species sequences generated in this study.

### Sequencing and sequence alignment

The sequences of the new species were subjected to standard BLAST searches in GenBank to find the most closely related sequences. All the sequences except those obtained from this study (Table 2), were retrieved from GenBank for phylogenetic analyses. Sequences were aligned using the online version of MAFFT v.7 (Katoh and Standley, 2013)^3^ and adjusted using BioEdit v.7.0.9 by hand (Hall, 1999) to minimize gaps and align properly. Ambiguous regions were excluded from the analyses and gaps were treated as missing data. The phylogeny website tool “ALTER” (Glez-Peña et al., 2010) was used to convert the Fasta alignment file to Phylip format for RAxML analysis and, AliView and PAUP 4.0b 10 were used to convert the Fasta alignment file to a Nexus file for Bayesian analysis (Swoford, 2003).

### Phylogenetic analyses

Maximum likelihood (ML) analysis was performed for both gene regions separately using RAxML-HPC2 v. 8.2.12 (Stamatakis, 2014) as implemented on the CIPRES portal (Miller et al., 2010), with the GTR + G model for both genes and 1,000 rapid bootstrap (BS) replicates. Since no supported conflict (BS ≥ 60%) was detected among the topologies, the four single-gene alignments were concatenated using SequenceMatrix (Vaidya et al., 2011).

Bayesian analysis was performed in MrBayes 3.2 (Ronquist et al., 2012) and the best-fit model of sequences evolution was estimated *via* MrModeltest 2.3 (Guindon and Gascuel, 2003; Nylander, 2004; Darriba et al., 2012). The Markov Chain Monte Carlo (MCMC) sampling approach was used to calculate posterior probabilities (PP; Rannala and Yang, 1996). Bayesian analysis of six simultaneous Markov chains was run for 10,000,000 generations and trees were sampled every 1,000 generations. The first 5,000 trees, representing the burn-in phase of the analyses, were discarded, while the remaining 1,500 trees were used for calculating posterior probabilities in the majority rule consensus tree (the critical value for the topological convergence diagnostic is 0.01).

Phylogenetic trees were visualized using FigTree v1.4.0,^4^ editing and typesetting using Adobe Illustrator CS5 (Adobe Systems Inc., United States). Sequences derived in this study were deposited in GenBank.^5^ The final sequence alignments and the phylogenetic trees are available at TreeBase (http://www.treebase.org, accession number: 29691).

## Results

### Phylogenetic analyses

**Figure 2:**
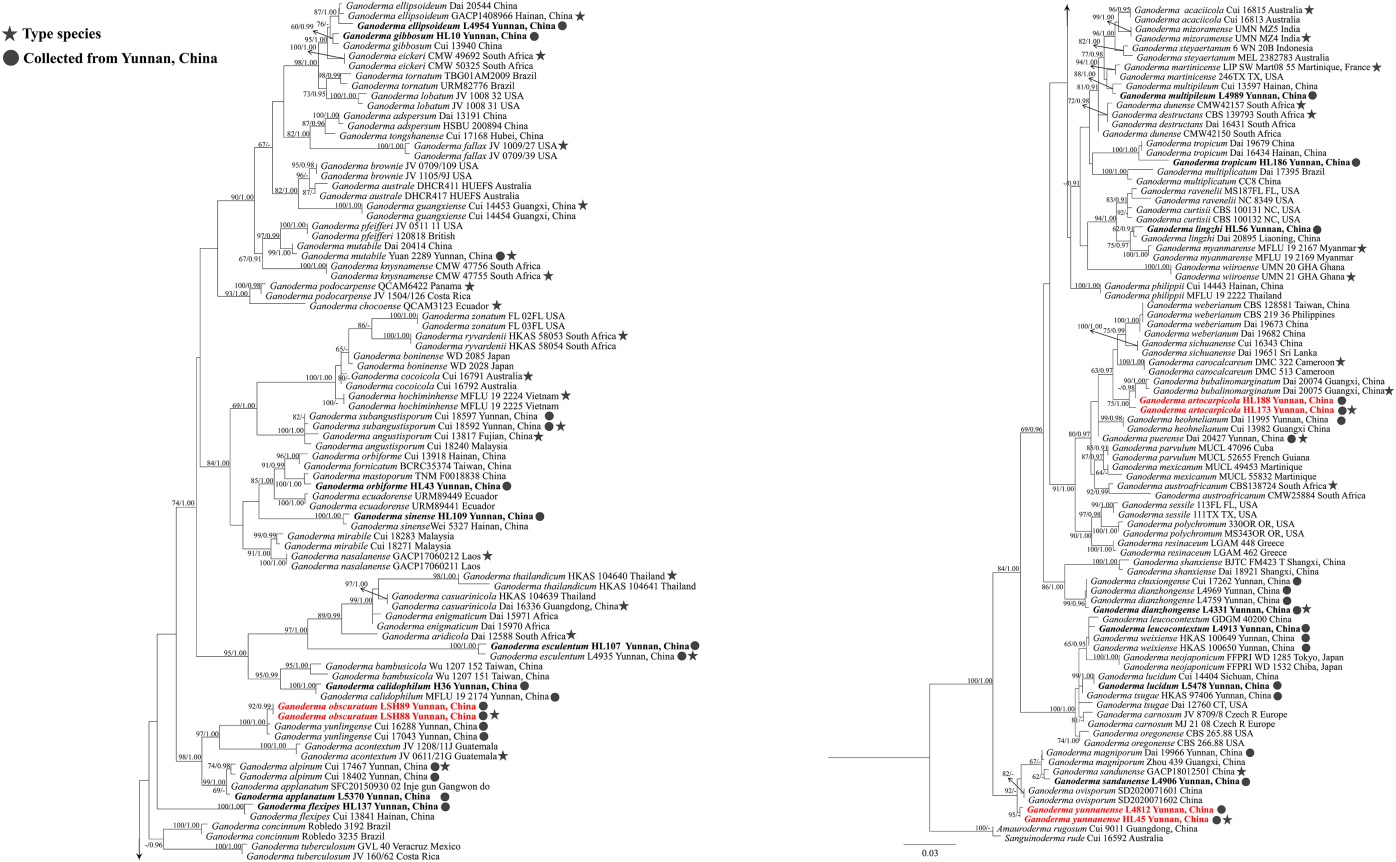
Maximum likelihood (ML) tree generated from a combined ITS + nrLSU sequence dataset. Bootstrap support values of maximum likelihood (ML) equal to or greater than 60% and Bayesian posterior probabilities (PP) equal to or greater than 0.90 are given above the nodes as “ML/PP.” New collections are indicated in black bold and new species are in red bold.

In this study, 71 *Ganoderma* sequences were newly generated from the specimens collected from YPC, and were deposited in GenBank (Table 2), i.e., 19 sequences of ITS, 21 sequences of nLSU, 18 sequences of tef1, and 13 sequences of rpb2. The combined two-gene dataset ITS + nrLSU (Figure 2) included sequences from 174 Ganodermataceae specimens representing 86 species. The dataset had an aligned length of 1,463 characters including gaps (ITS: 1–611; nrLSU: 612–1,463), of which *Amauroderma rugosum* Cui 9,011 and *Sanguinoderma rude* Cui 16,592 as the outgroup taxa (Figure 2; Sun et al., 2020, 2022). The Maximum likelihood analysis based on the concatenated ITS + nLSU dataset resulted in a similar topology as Bayesian Inference analysis. The RAxML analysis of the combined dataset yielded the best scoring tree with a final ML likelihood value of −8472.680716 (Figure 2). The matrix had 475 distinct alignment patterns, with 33.97% undetermined characters or gaps. Estimated base frequencies were as follows: A = 0.230978, C = 0.222798, G = 0.276648, T = 0.269576; substitution rates AC = 1.230871, AG = 4.648437, AT = 1.401201, CG = 1.020212, CT = 9.538270, GT = 1.000000, *α* = 0.177171, Tree-Length: 1.586199. The best model for the ITS + nLSU dataset estimated and applied in the Bayesian analysis was HKY + I + G for ITS and GTR + I + G for nrLSU.

**Figure 3:**
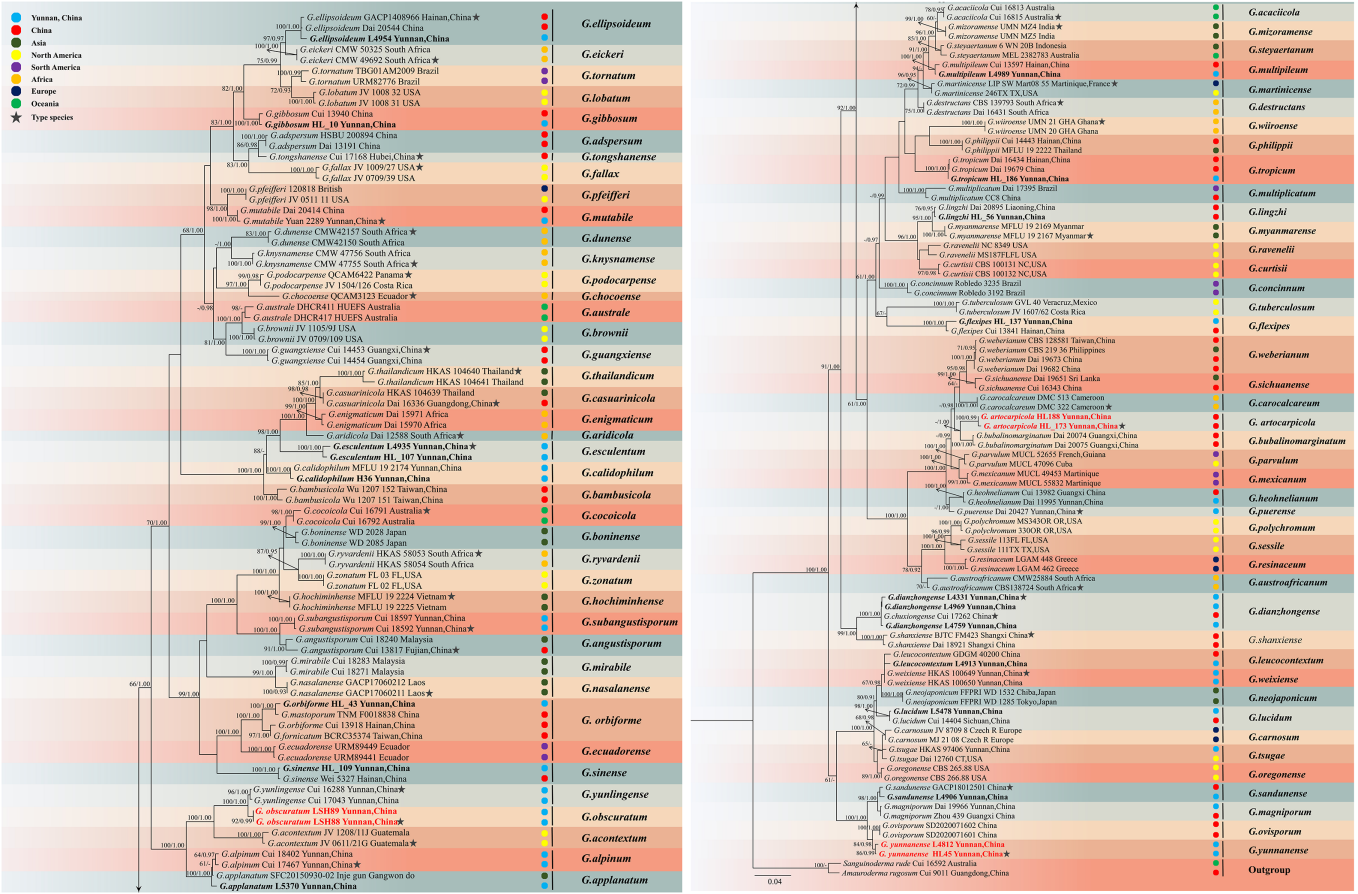
Maximum likelihood (ML) tree generated from a combined ITS + nrLSU + TEF1-α + RPB2 sequence dataset. Bootstrap support values with a maximum likelihood (ML) equal to or greater than 60% and Bayesian posterior probabilities (PP) equal to or greater than 0.90 given above the nodes as “ML/PP.” New collections are indicated in black bold while new species are in red bold.

The dataset is composed of combined ITS + nrLSU + TEF1-α + RPB2 sequences data from 174 specimens, representing 86 taxa in *Ganodermataceae*. The aligned dataset comprised 2,995 characters including gaps (ITS: 1–611; nrLSU: 612–1,463; TEF1-α: 1,464–2002; RPB2: 2,003–2,663). Tree topology of the maximum likelihood analysis and Bayesian analysis is similar. The RAxML analysis of the combined dataset yielded the best scoring tree with a final ML likelihood value of −33599.741722 (Figure 3). The matrix had 1,087 distinct alignment patterns, with 36.13% undetermined characters or gaps. Estimated base frequencies were as follows: A = 0.223924, C = 0.253042, G = 0.274308, T = 0.248726; substitution rates AC = 1.353439, AG = 6.944619, AT = 1.408316, CG = 1.653377, CT = 9.659772, GT = 1.000000, *α* = 0.194286, Tree-Length: 1.880697. Best model for the ITS + nLSU + TEF1-α + RPB2 dataset estimated and applied in the Bayesian analysis were HKY + I + G for ITS [Lset nst = 2, rates = invgamma; Prset statefreqpr = Dirichlet (1,1,1,1)] and GTR + I + G for nrLSU, TEF1-α and RPB2 [Lset nst = 6, rates = invgamma; Prset statefreqpr = Dirichlet (1,1,1,1)]. ML and BI analyses generated nearly identical tree topologies with minimal variations in statistical support values. Thus, only a ML tree is shown. Bootstrap support values in maximum likelihood (ML) equal to or greater than 60%, and Bayesian posterior probabilities (PP) equal to or greater than 0.90 are given above the nodes (Figures 2, 3).

The multigene phylogenetic analyses showed that 18 of our new specimens are nested in *Ganoderma*, of which three are described as new species. *Ganoderma artocarpicola* sp. nov. was sister to *G. bubalinomarginatum* B.K. Cui, J.H. Xing and Y.F. Sun with high statistical supports (−ML/1.00PP, Figure 3). *Ganoderma obscuratum* sp. nov. clustered as a sister clade with *G. yunlingense* B.K. Cui, J.H. Xing & Y.F. Sun and *G. acontextum* B.K. Cui, J.H. Xing & Vlasák with high statistical support (100%ML/1.00PP, Figure 3). The third species, *G. yunnanense* sp. nov. closely clustered with *G. ovisporum* H.D. Yang, T.C. Wen, *G. magniporum* J.D. Zhao & X.Q. Zhang and *G. sandunense* Hapuar., T.C. Wen and K.D. Hyde with high statistical support (100%ML/1.00PP), and a distinct lineage.

### Taxonomy

**Figure 4:**
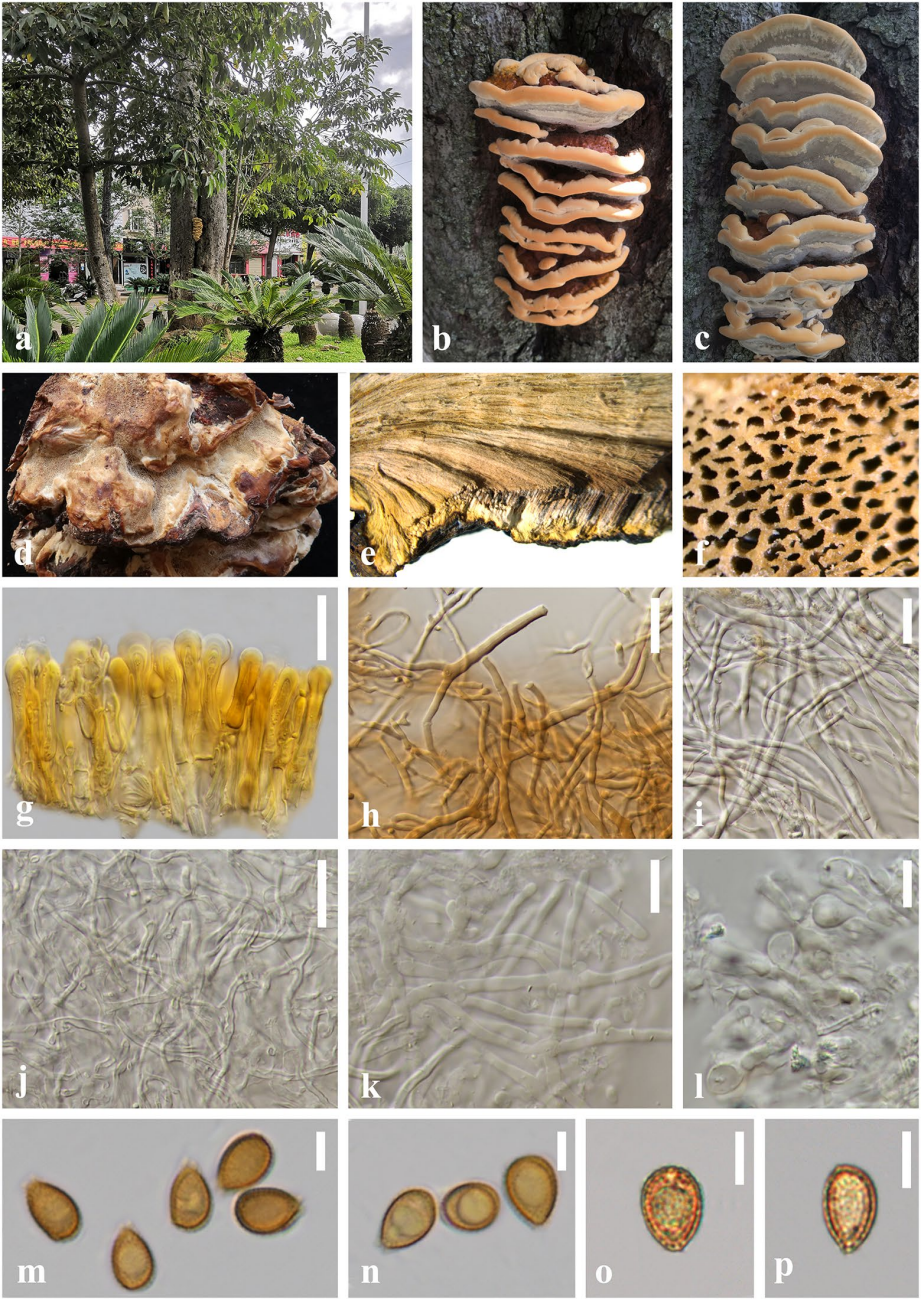
*Ganoderma artocarpicola* (HKAS 123782, holotype) **(A–C)** Basidiomata *in situ* on *Artocarpus pithecogallus* living tree. **(D)** Lower surface. **(E)** Transverse section of pileus. **(F)** Pore surface. **(G)** Sections of pileipellis. **(H,I)** Skeletal hyphae from context. **(J)** Binding hyphae from context. **(K)** Generative hyphae from tubes. **(L)** Basidia and basidioles. **(M–P)** Basidiospores. Scale bars: **(G–J)** = 20 μm, **(K,L)** = 10 μm, **(M–P)** = 5 μm. Photographs were taken by JH.

*Ganoderma artocarpicola* J. He and S.H. Li, sp. nov. (Figure 4).

MycoBank number: MB845720

*Diagnosis: Ganoderma artocarpicola* is characterized by its sessile and concrescent basidiomata, reddish brown to yellowish brown pileus surface with shallow concentric furrows and radial rugose, heterogeneous context, wavy margin and ellipsoid to ovoid basidiospores (8.0–10.5 × 5.0–7.5 μm).

*Etymology:* The epithet ‘*artocarpicola*’ refers to the host tree genus *Artocarpus*.

*Holotype:* CHINA. Yunnan Province., Lincang City, Yongde County (24°54′51″N, 99°15′31″E), on living tree of *Artocarpus pithecogallus*, alt. 1,506 m, Jun He, 21 September 2021, HL188 (HKAS 123782).

*Description:* Basidiomata: annual, sessile and broadly attached, usually concrescent, woody hard. Pileus: imbricate, flabelliform to reniform, slightly convex to applanate, projecting up to 9 cm, 8 cm wide and 2 cm thick at the base. Pileus surface reddish brown (9E8) to yellowish brown (5C7), weakly to strongly laccate, with shallowly concentric furrows and radial rugose, concentrically zonate or azonate. Margin: buff (1A3) to grayish orange (6D8), entire, obtuse, irregularly wavy. Context: up to 1.8 cm thick, heterogeneous, the upper layer greyish white(2B1), the lower layer cinnamon brown (6D7) to chestnut brown (8E5), without black melanoid lines, hard corky and fibrous. Tubes: 0.2–0.5 cm long, dark brown (6E8), woody hard, unstratified. Pores: 5–7 per mm, circular to angular, dissepiments thick, entire; pores surface cream (2B2) to greyish white (2B1) when fresh, golden grey to greyish brown when bruising and drying.

Hyphal system trimitic: generative hyphae 2.0–3.5 μm in diameter, colorless, thin-walled, with clamp connections; skeletal hyphae 2.0–5.0 μm in diameter, thick-walled with a narrow lumen to sub-solid, arboriform and flexuous, pale yellow to yellowish brown; binding hyphae 1.5–3.0 μm in diameter, thick-walled, frequently branched, interwoven, colorless, scarce; all the hyphae IKI–, CB+; tissues darkening in KOH.

Pileipellis: a crustohymeniderm, cells 35–50 × 5–10 μm, thick-walled to sub-solid, apical cells clavate, inflated and flexuous, pale yellow to golden yellow, without granulations in the apex, moderately amyloid at maturity.

Basidiospores: ellipsoid to ovoid, not obviously truncated, with apical germ pore, yellowish to golden yellow, IKI–, CB+, inamyloid; double walled with slightly thick walls, exospore wall smooth, endospore wall with inconspicuous spinules; (60/3/2) 8.0 (8.5)–*9.3*–10.0 (10.5) × 5.0 (5.5)–*6.2*–7.0 (7.5) μm, *L* = 9.25 μm, *W* = 6.20 μm, *Q* = (1.23) 1.31–*1.50*–1.72 (1.78), *Q*_m_ = 1.50 ± 0.14 (including myxosporium). Basidia: barrel-shaped to utriform, colorless, with a clamp connection and four sterigmata, thin-walled, 10–15 × 5–9 μm; basidioles pear-shaped to fusiform, colorless, thin-walled, 8–10 × 4–7 μm.

*Additional specimen examined:* China, Yunnan Province, Lincang City, Yongde County, Dedang Town (24°01′12″N, 99°15′34″E), on a living tree of *Artocarpus pithecogallus*, alt. 1,484 m, Qian-Qiu Luo, 22 August 2021, HL173 (HKAS 123783).

*Notes:* In the phylogenetic analyses, *G. artocarpicola* is sister to *G. bubalinomarginatum*, which was described from the southwest Guangxi Province in China (Figure 3; Sun et al., 2022). Morphologically, both species share similar characteristics of the connate and sessile basidiomata, reddish brown to yellowish brown pileus surface, and non-stratified tubes. However, *G. bubalinomarginatum* differs from *G. artocarpicola* in having buff and obtuse pileus margin, smaller basidiospores (7.0–8.8 × 4.3–5.8 μm), and larger basidia (15–22 × 7–11 μm, Sun et al., 2022).

*Ganoderma weberianum* and *G. artocarpicola* are similar in having imbricate, sessile and hard basidiomata. However, *G. weberianum* has a pale-yellow pore surface when dry, homogeneous greyish brown context, smaller basidiospores (6.0–7.0 × 4.0–6.0 μm), and longer pileipellis (60.0–90.0 × 6.0–12.0 μm, Steyaert, 1972; Pan and Dai, 2001). In addition, the pileus of *G. weberianum* is more laccate than *G*. *artocarpicola*. The comparison of the ITS sequences of *G. weberianum* and *G. artocarpicola* showed 2.12% (13/614 bp) nucleotide differences.

**Figure 5:**
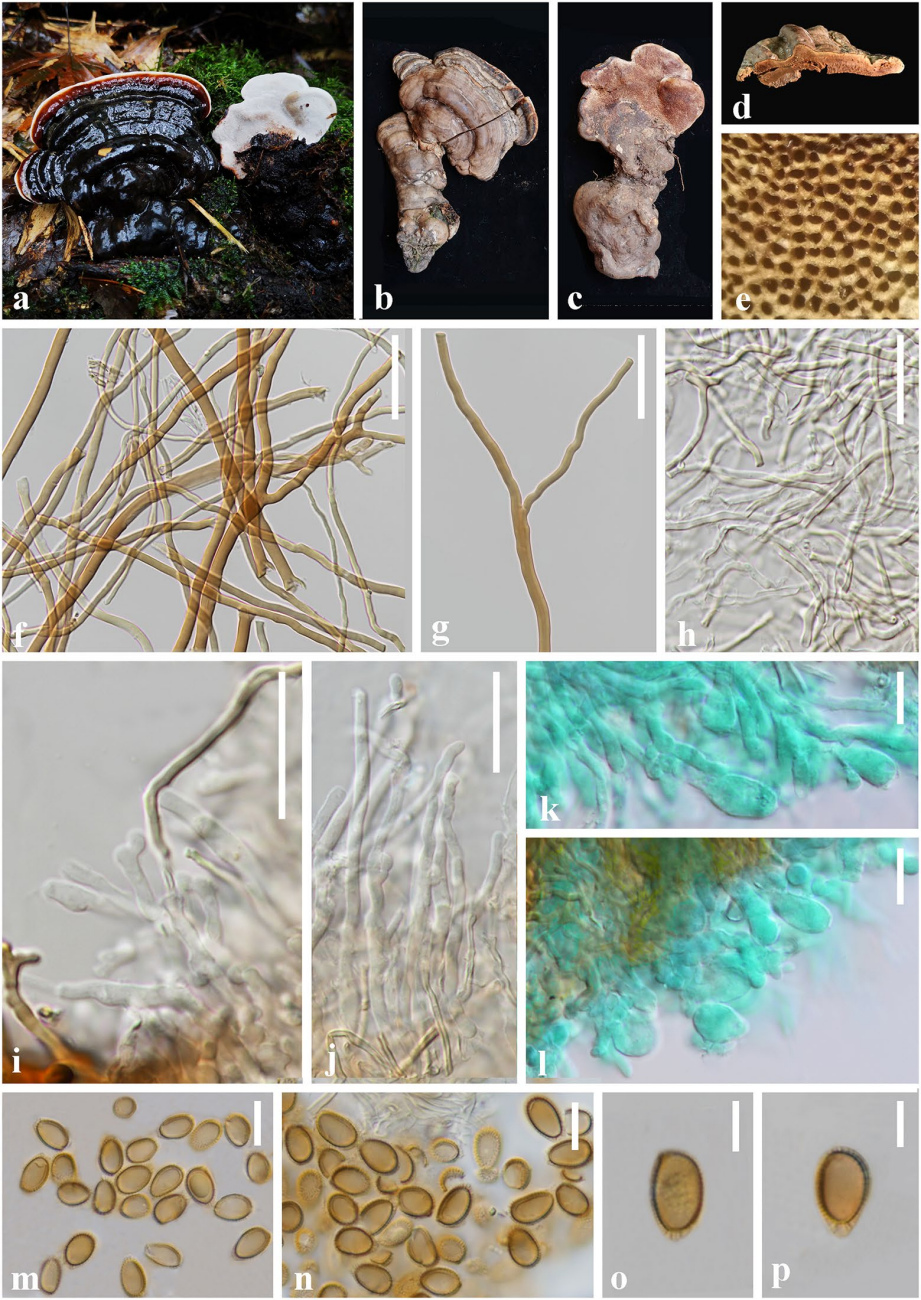
*Ganoderma obscuratum* (HKAS 123786, holotype) **(A–C)** Basidiomata. **(D)** Transverse section of pileus. **(E)** Pore surface. **(F,G)** Skeletal hyphae from context. **(H)** Binding hyphae from context. **(I,J)** Generative hyphae from tubes. **(K,L)** Basidia and basidioles. **(M–P)** Basidiospores. Scale bars: **(F,G)** = 30 μm, **(H–N)** = 10 μm, **(O,P)** = 5 μm. Photographs were taken by XH.

*Ganoderma obscuratum* J. He and S.H. Li, sp. nov. (Figure 5).

MycoBank number: MB845721

*Diagnosis: Ganoderma obscuratum* is characterized by its small and dorso-laterally stipitate basidiomata, dark brown to greyish brown and laccate pileus surface, small pores (6–9 per mm), corky context, and almond-shaped to narrow ellipsoid basidiospores (8.0–9.5 × 4.5–5.5 μm).

*Etymology:* The epithet ‘*obscuratum*’ refers to the obscure pileus surface when dry.

*Holotype:* CHINA. Yunnan Province., Zhaotong City, Yiliang County (104°14′55″E, 27°47′56″N), on a dead tree of *Acer* sp. alt. 1,859 m, Shu-Hong Li, 12 August 2019, Lsh88 (HKAS 123786).

*Description: Basidiomata:* annual, sessile to substipitate, coriaceous to woody hard, light in weight. Pileus: single, flabelliform to reniform or shell-shaped, applanate, projecting up to 6 cm, 4.5 cm wide and 1 cm thick at the base. Pileus surface dark brown (8E8) when fresh becoming greyish brown (7E8) when dry, and covered by a thin hard crust, laccate, glabrous and shiny, with dense concentric furrows. Margin: buff (8B2) to generally concolorous, entire, subacute to obtuse, slightly wavy, cracked when dry. Context: up to 0.7 cm thick, homogeneous, yellowish brown (5D5) to chestnut brown (6E8), with black melanoid lines, hard corky. Tubes: 0.2–0.4 cm long, concolorous with the base of the context, corky, unstratified. Pores: 6–9 per mm, circular, dissepiments slightly thick, entire; pores surface white to greyish white (2B1) when fresh, pale brown (6D6) to dark brown (7E7) when bruising and drying. Stipe: up to 6.5 cm long and 2.2 cm diam, flattened to cylindrical, fibrous to spongy, concolorous with pileus surface.

Hyphal system trimitic: generative hyphae 2.0–4.0 μm in diameter, colorless, thin-walled, with clamps connections; skeletal hyphae 2.0–8.0 μm in diameter, thick-walled with a wide to narrow lumen or sub-solid, arboriform with few branches, yellowish brown to golden yellow; binding hyphae 1.0–3.0 μm in diameter, thick-walled, branched and flexuous, colorless to pale yellow, scarce; all the hyphae IKI–, CB+; tissues darkening in KOH.

Basidiospores: almond-shaped to narrow ellipsoid, apex subacute, with apical germ pore, yellowish to yellowish brown, IKI–, CB+, inamyloid; double-walled with moderately thick walls, exospore wall smooth, endospore wall with inconspicuous spinules; (40/2/2; 8.0) 8.5–*9.0*–9.0 (9.5) × 4.5–*5.2*–5.0 (5.5) μm, L = 9.09 μm, W = 5.22 μm, *Q* = (1.58) 1.61–*1.75*–1.87 (2.08), *Q*_m_ = 1.75 ± 0.11 (including myxosporium). Basidia: broadly clavate, colorless, with a clamp connection and four sterigmata, thin-walled, 15–25 × 5–9 μm; basidioles in shape like the basidia, colorless, thin-walled, 10–21 × 4–8 μm.

*Additional specimens examined:* China, Yunnan Province, Zhaotong City, Yiliang County, Xiaocaoba Town (104°14′18″E, 27°47′59″N), on a dead tree of *Acer* sp., alt. 1,905 m, Shu-Hong Li, 12 August 2019, Lsh89 (HKAS 123772).

*Notes:* Phylogenetic analyses showed that *Ganoderma obscuratum* clusters as a sister taxon to *G. yunlingense* with good statistical support (100% ML/1.00 PP, Figure 3). Morphologically, *G. obscuratum* differs from *G. yunlingense* by having thin basidiomata, dark brown and laccate pileus surface when fresh, homogeneous context and non-stratified tubes, smaller pores (6–9 per mm), and narrow ellipsoid basidiospores with spinules on the endospore wall (Sun et al., 2022).

*Ganoderma alpinum* described from Yunnan Province is morphologically similar to *G. obscuratum* by having the hard basidiomata with greyish brown pileus surface, homogeneous context and non-stratified tubes. However, *G. alpinum* differs by the larger pores (5–7per mm), and smaller basidiospores (6.2–7.8 × 4–5.5 μm, Sun et al., 2022). *Ganoderma applanatum* also has sessile basidiomata and homogeneous context, but it differs from G. *obscuratum* by having a perennial basidiomata with pale pileus surface and smaller basidiospores (5–8 × 4–6 μm, Moncalvo and Ryvarden, 1997; Hapuarachchi et al., 2019; Sun et al., 2022). Besides, *G. applanatum* and *G. obscuratum* were well separated in the phylogenetic analyses (Figure 3).

**Figure 6:**
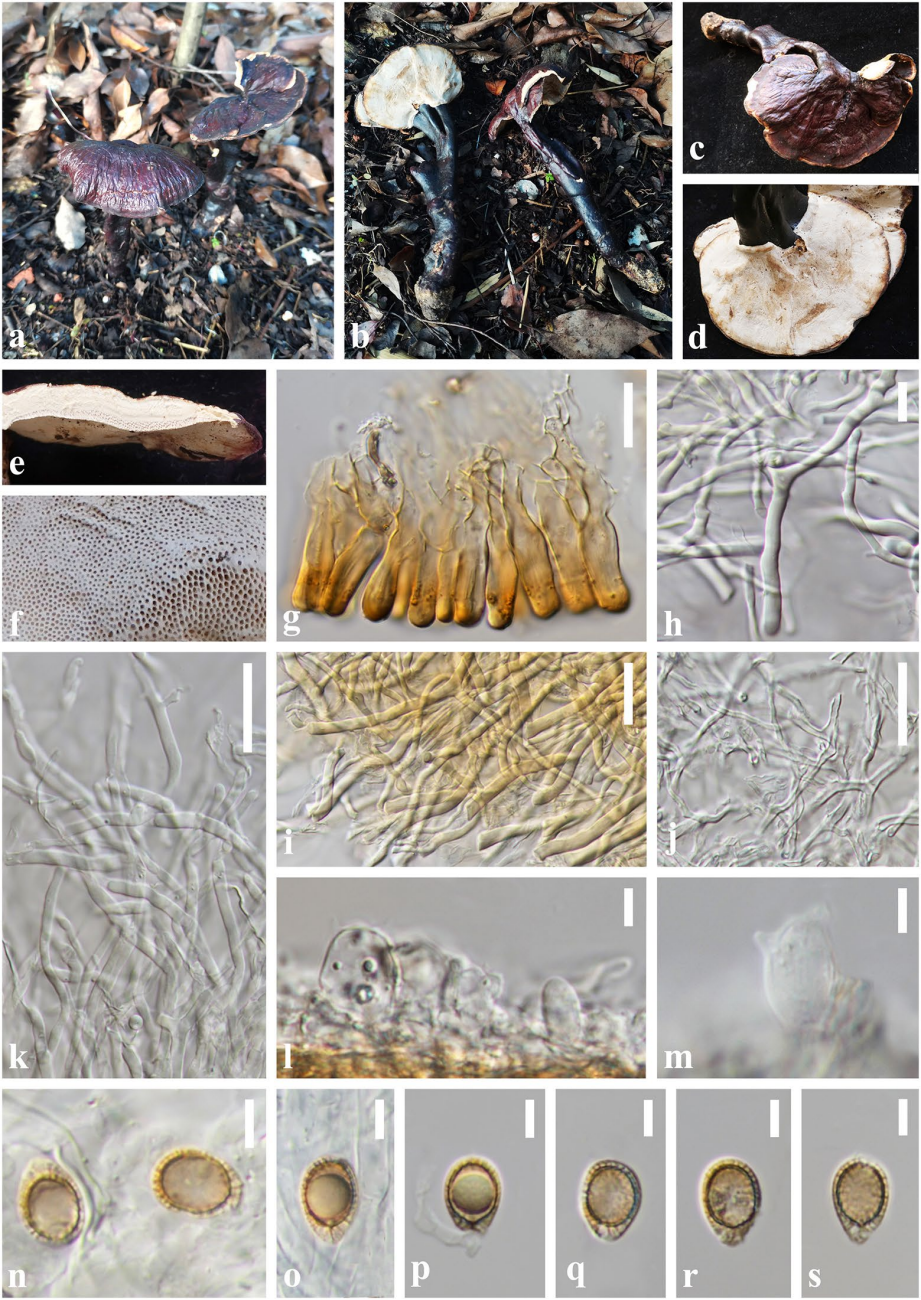
*Ganoderma yunnanense* (HKAS 123771, holotype) **(A,B)** Basidiomata. **(C)** Upper surface. **(D)** Lower surface. **(E)** Transverse section of pileus. **(F)** Pore surface. **(G)** Sections of pileipellis. **(H,I)** Skeletal hyphae from context. **(J)** Binding hyphae from context. **(K)** Generative hyphae from tubes. **(L,M)** Basidia and basidioles. **(N–S)** Basidiospores. Scale bars: **(I–K)** = 20 μm, **(G,H)** = 10 μm, **(L–S)** = 5 μm. Photographs were taken by JH.

*Ganoderma yunnanense* J. He and S.H. Li, sp. nov. (Figure 6).

MycoBank number: MB845722

*Diagnosis: Ganoderma yunnanense* is characterized by its centrally to laterally stipitate basidiomata with reddish brown to violet brown and strongly laccate pileus surface, cream color pore surface and context, and broadly ellipsoid basidiospores (8.0–12.5 × 7.0–9.0 μm).

*Etymology:* The epithet ‘*yunnanense*’ refers to Yunnan Province from where the holotype was collected.

*Holotype:* CHINA. Yunnan Province, Puer City, Jingdong County, Wuliang Mountains (100°48′48″E, 24°19′36″N), on a rotten broad-leaved tree, alt. 2,129 m, Song-Ming Tang, 8 August 2021, HL45 (HKAS 123771).

*Description. Basidiomata:* annual, centrally to laterally stipitate, hard corky. Pileus: single, flabelliform to reniform or suborbicular, projecting up to 9 cm, 6.5 cm wide and 0.5 cm thick at base. Pileus surface reddish brown (10F8) to violet brown (11F8), weakly to strongly laccate, glossy, with shallowly concentric furrows and radial rugose. Margin: pale yellow (3B2) to concolorous, entire, acute, incurved when dry. Context: up to 0.3 cm thick, homogeneous, white to cream (1B2), fibrous, corky, without black melanoid lines. Tubes: 0.1–0.2 cm long, concolorous with the base of the context, corky, unstratified. Pores: 4–6 per mm, round to angular, dissepiments thick, entire; pore surface white when fresh, lead grey (3B1) when bruising and drying. Stipe: 15.0–17.5 × 1.0–2.0 cm, dorsally lateral to nearly dorsal, cylindrical and solid, concolorous with pileus surface, strongly laccate, fibrous to woody.

Hyphal system trimitic: generative hyphae 2.0–3.0 μm in diameter, colorless, thin-walled, with clamps connections; skeletal hyphae 2.0–6.0 μm in diameter, subthick-walled to solid, non-septate, arboriform with few branches, colorless to pale yellow; binding hyphae 1.5–3.0 μm in diameter, thick-walled, frequently branched and flexuous, colorless, scarce; all the hyphae IKI–, CB+; tissues darkening in KOH.

Pileipellis: a crustohymeniderm, composed of a palisade of vertical, cells 23–40 × 6–9 μm, slightly thick-walled, clavate to cylindrical, slightly inflated, straw yellow to golden-yellow, granulations in the apex, moderately clavate to cylindrical amyloid at maturity.

Basidiospores: broadly ellipsoid to ellipsoid, apex not obviously truncated, with apical germ pore, yellowish to pale yellowish brown, IKI–, CB+, inamyloid; double-walled with distinctly thick walls, exospore wall smooth, endospore walls with inter-wall pillars; (40/2/2) (8.0) 9.0–*10.7*–12.0 (12.5) × 7.0–*7.6*–8.0 (8.5) μm, *Q* = (1.10) 1.25–*1.41*–1.55 (1.60), *Q*_m_ = 1.41 ± 0.12 (including myxosporium). Basidia: widely clavate to barrel-shaped, colorless, with a clamp connection and four sterigmata, thin-walled, 15–18 × 8–11 μm; basidioles clavate, colorless, thin-walled, 10–14 × 6–9 μm.

*Additional specimens examined:* China, Yunnan Province., Puer City, Jingdong Co-unty, Ailao Mountains (101°01′29″E, 24°30′03 N), on a rotten broad-leaved tree, alt. 2,326 m, Jun He, 4 August 2019, L4812 (HKAS 123769).

*Notes:* Our multi-locus phylogenetic analyses show that *Ganoderma yunnanense* is sister to *G. ovisporum* with high statistical support (84% ML/0.98 PP, Figure 3), and together they group with *G. sandunense* and *G. magniporum* (Zhao et al., 1984; Hapuarachchi et al., 2019; Yang et al., 2022). *Ganoderma yunnanense* resembles *G. ovisporum* in having reddish-brown pileus and broadly ellipsoid basidiospores. However, *G. ovisporum* has heterogeneous context, shorter pileipellis cells (18–29 × 6–11 μm) and larger basidiospores (12.5–15.5 × 9.0–11.5 μm, Yang et al., 2022). Moreover, *Ganoderma sandunens* has a larger basidiospores (10.8–15.7 × 8.6–12.5 μm) and thicker context than those of *G. yunnanense* (Hapuarachchi et al., 2019; Yang et al., 2022). *Ganoderma magniporum* can be easily distinguished from *G. yunnanense* by the larger pores (2–2.5 per mm), black-brown to black pileus surface and ovoid basidiospores with truncated apex (8.7–10.4 × 5.2–7.0 μm, Zhao et al., 1984).

Morphologically, *G. yunnanense* resembles *G. leucocontextum* by white pore surface and context. However, *G. leucocontextum* has red to red brown pileus surface, white to yellowish margin, shorter stipe (5–10 cm) and broadly ellipsoid basidiospores with truncated apex (8.0–12.5 × 5.5–9.0 μm, Li et al., 2015). Among the species in the *G. lucidum* complex, *G. yunnanense* looks very similar to *G. tsugae* and *G. weixiense* morphologically, although they can be easily distinguished by phylogenetic analyses and ecological distribution (Murrill, 1902; Ye et al., 2019).

In addition, *G. yunnanense* also shares similarities with *G. dianzhongense* but the latter has dark-brown to putty context and wider pileipellis cells than those of *G. yunnanense*. The nucleotide comparison of ITS sequences of *G. yunnanense* and *G. dianzhongense* revealed 26 bp (26/614 bp, 4.23%) nucleotides differences (He et al., 2021).

### Key to the species of *Ganoderma* in Yunnan Province, China

**Table:** 

1. Pileal surface non-laccate	2
1*. Pileal surface laccate	11
2. Pileus imbricate, margin lacerated like petals	*G. puerense*
2*. Pileus solitary, margin entire	3
3. Basidiospores subglobose	*G. hoehnelianum*
3*. Basidiospores broadly ellipsoid to ellipsoid or ovoid	4
4. Tubes stratified	5
4*. Tubes non-stratified	6
5. Context homogeneous; basidiospores 5.5–7 × 4.1–5.2 μm	*G. applanatum*
5*. Context heterogeneous; basidiospores 7–12 × 5–8 μm	*G. australe*
6. Pores > 6 per mm	*G. obscuratum*
6*. Pores < 6 per mm	7
7. Context without black melanoid lines; apical cells in cuticle branched	*G. ellipsoideum*
7*. Context with black melanoid lines; apical cells in cuticle unbranched	8
8. Distributed in higher altitudes	*G. alpinum*
8*. Distributed in lower altitudes	9
9. Apical cells in cuticle irregularly branched or with protuberances	*G. williamsianum*
9*. Apical cells in cuticle unbranched or without protuberances	10
10. Pileus surface reddish brown to greyish brown, pores angular	*G. gibbosum*
10*. Pileus surface greyish brown to grey, pores circular	*G. yunlingense*
11. Basidiomata sessile	12
11*. Basidiomata stipitate or with constricted short stipe	14
12. Apical cells in cuticle irregularly branched or with protuberances	*G. mutabile*
12*. Apical cells in cuticle unbranched or without protuberances	13
13. Pileus surface reddish brown to yellowish brown; basidiospores > 8 μm in length	*G. artocarpiccola*
13*. Pileus surface pale brown to purplish black; basidiospores < 8 μm in length	*G. philippii*
14. Pores < 3 per mm	*G. magniporum*
14*. Pores > 3 per mm	15
15. Pileus surface dark-red to nearly black	16
15*. Pileus surface pale brown to yellowish brown or reddish brown	20
16. Stipe short or constricted at base, < 4 cm in length	17
16*. Stipe obviously long, > 4 cm in length	18
17. Basidiospores subglobose to broadly ellipsoid, < 6 μm in width	*G. weberianum*
17*. Basidiospores ellipsoid to ovoid, > 6 μm in width	*G. orbiforme*
18. Basidiomata central stipitate; basidiospores truncated	*G. sanduense*
18*. Basidiomata laterally stipitate; basidiospores not obviously truncated	19
19. Context homogeneous, pores 5–6 per mm; basidiospores 10.3–13.1 × 5.0–7.3 μm	*G. subangustisporum*
19*. Context heterogeneous, pores 3–5 per mm; basidiospores 11.0–13.7 × 7.0–8.8 μm	*G. sinense*
20. Pore surface yellowish to buff when fresh	21
20*. Pore surface white to greyish white or cream when fresh	22
21. Pileus surface oxblood red to violet brown; basidiospores > 7μm in width	*G. dianzhongense*
21*. Pileus surface reddish brown to yellowish brown; basidiospores < 7 μm in width	*G. lingzhi*
22. Distributed in temperate areas	23
22*. Distributed in tropical areas	28
23. Growing on coniferous trees	*G. tsugae*
23*. Growing on broad-leaf trees	24
24. Basidiospores < 5 μm in width	*G. weixiense*
24*. Basidiospores > 5 μm in width	25
25. Context with black melanoid lines	*G. sichuanense*
25*. Context without black melanoid lines	26
26. Context heterogeneous, buff to dark brown	*G. lucidum*
26*. Context homogeneous; white to cream or greyish white	27
27. Pileus surface red to red brown; basidiospores truncated	*G. leucocontextum*
27*. Pileus surface violet brown; basidiospores not obviously truncated	*G. yunnanense*
28. Stipe short or constricted at base, < 6 cm in length	*G. tropicum*
28*. Stipe obviously long, > 6 cm in length	29
29. Pileus imbricate, upper surface orange yellow to orange red	*G. multipileum*
29*. Pileus solitary, upper surface reddish brown to black brown	30
30. Growth on broad-leaved forests	*G. flexipes*
30*. Growth on bamboo forests.	31
31. Context heterogeneous, pores 4–6 per mm; basidiospores 8.0–10.5 × 5.5–9.1 μm	*G. calidophilum*
31*. Context homogeneous, pores 5–8 per mm; basidiospores 8.0–12.5 × 5.0–8.0 μm	*G. esculentum*

## Discussion

Sun et al. (2022) revealed the species diversity, taxonomy and phylogeny of Ganodermataceae with emphasis on Chinese collections, which showed that 40 species of *Ganoderma* in China were confirmed by morphology and DNA sequence data. Among the 40 species, five new species of *Ganoderma* were discovered in YPC, namely *G. alpinum*, *G. chuxiongense*, *G. puerense*, *G. subangustisporum*, and *G. yunlingense*. Besides, Sun et al. (2022) summarized known species of *Ganoderma* in YPC *viz. G. ellipsoideum*、*G. flexipes*、G*. hoehnelianum*、*G. lingzhi*、and *G. magniporum*. However, results of our research showed that *Ganoderma chuxiongense* and *G. dianzhongense* are similar in morphology and phylogeny, and based on the time priority, *G. chuxiongense* is considered as a synonym of *G. dianzhongense*. In consideration of the authors’ contributions, it is suggested to use the sample Cui 17,262 (BJFC034120) as a paratype of *Ganoderma dianzhongense* (He et al., 2021; Sun et al., 2022).

To date, 25 species of *Ganoderma* have been recorded in YPC (Cao et al., 2012; Ye et al., 2019; He et al., 2021; Sun et al., 2022), however, the species diversity of *Ganoderma* is still not well known, especially in the subtropical and tropical areas. According to our survey of different sample sites in Yunnan Province from 2016 to 2021, a total of 268 samples of *Ganoderma* were collected. Based on comprehensive morphological characteristics and phylogenetic evidence, we report 15 known species of *Ganoderma* from YPC *viz. Ganoderma applanatum*, *G. calidophilum*, *G. dianzhongense*, *G. ellipsoideum*, *G. esculentum*, *G. flexipes*, *G. gibbosum*, *G. leucocontextum*, *G. lingzhi*, *G. lucidum*, *G. multipileum*, *G. orbiforme*, *G. sandunense*, *G. sinense* and *G. tropicum*. In addition, three new species *viz. G. artocarpicola*, *G. obscuratum* and *G. yunnanense* are proposed in this study. Up to now, 183 species of *Ganoderma* have been described all over the world, of which 42 species have been recorded in China (Wu et al., 2020; Sun et al., 2022; Yang et al., 2022). The discovery of three new species of *Ganoderma* in this study raises the known *Ganoderma* species in Yunnan Province to 32, accounting for 71.11% of the known *Ganoderma* species in China. Thus, Yunnan Province can be considered as one of the biodiversity center hot spots for *Ganoderma*.

A checklist of *Ganoderma* in YPC is given in Table 3. In addition, a key to *Ganoderma* in YPC is also provided. This paper enriches the knowledge of *Ganoderma* in YPC, and it is likely that more new taxa will be discovered in the future with extensive sampling in different areas and comprehensive molecular analyses.

**Table 3:** Species, hosts, and geographical locations and corresponding references of *Ganoderma* in Yunnan Province, China.

Species	Host plant	Location	References
*Ganoderma alpinum*	*Populus* sp.	Shangri-La	Sun et al. (2022)
*G. applanatum*	*Eriobotrya japonica*	Nujiang Prefecture	This study
*G. artocarpicola*	*Artocarpus* sp.	Lincang City	This study
*G. australe*	*Fagus* sp.	Kunming City	Luangharn et al. (2021)
*G. calidophilum*	On bamboo roots	Dehong Prefecture	This study, He et al. (2021)
*G. dianzhongense*	*Cyclobalanopsis glauca*	Central Yunnan Province	This study, He et al. (2021)
*G. ellipsoideum*	Broad-leaved tree	Honghe Prefecture	This study
*G. esculentum*	*Bambusa intermedia*	Honghe Prefecture	He et al. (2021)
*G. flexipes*	*Castanopsis fargesii*	Puer City	This study
*G. gibbosum*	*Carya cathayensis*	Zhaotong City	This study
*G. hoehnelianum*	Broad-leaved tree	Jinghong City	Wang and Wu (2010), Xing (2019)
*G. leucocontextum*	*Cyclobalanopsis glauca*	Dali Prefecture	This study
*G. lingzhi*	Broad-leaved tree	Kunming City	This study
*G. lucidum*	*Quercus* sp.	Chuxiong Prefecture	This study
*G. magniporum*	Broad-leaved tree	Yunnan Province	Sun et al. (2022)
*G. multipileum*	*Acacia farnesiana*	Yuxi City	This study
*G. mutabile*	Angiosperm tree	Chuxiong Prefecture	Cao (2013)
*G. obscuratum*	*Acer* sp.	Zhaotong City	This study
*G. orbiforme*	*Quercus acutissima*	Honghe Prefecture	This study
*G. philippii*	*Hevea brasiliensis*	Sipsongpanna	Zhao (1988)
*G. puerense*	*Cinnamomum* sp.	Puer City	Sun et al. (2022)
*G. sandunense*	*Quercus* sp.	Honghe Prefecture	This study
*G. sichuanense*	*Cyclobalanopsis* sp.	Kunming City	Luangharn et al. (2021)
*G. sinense*	Broad-leaved tree	Wenshan Prefecture	This study
*G. subangustisporum*	Angiosperm tree	Wenshan Prefecture	Sun et al. (2022)
*G. tsugae*	*Picea* sp.	Kunming City	Luangharn et al. (2021)
*G. tropicum*	*Acacia* sp.	Puer City	This study
*G. weixiensis*	coniferous forest	Diqing Prefecture	Ye et al. (2019)
*G. weberianum*	*Ficus* sp.	Jinghong City	Pan and Dai (2001)
*G. williamsianum*	Broad-leaved tree	Puer City	Cao and Yuan (2013)
*G. yunlingense*	*Quercus* sp.	Nujiang Prefecture	Sun et al. (2022)
*G. yunnanense*	Broad-leaved trees	Puer City	This study

## Data availability statement

The original contributions presented in the study are included in the article/supplementary material, further inquiries can be directed to the corresponding author.

## Author contributions

S-HL and Z-LL: conceptualization. JH: methodology, formal analysis, data curation, and writing—original draft preparation. JH and XH: investigation. S-HL and Z-ZL: resources. K-YN, S-MT, E-XL, H-ML, and S-HL: writing—review and editing. S-HL: funding acquisition. All authors contributed to the article and approved the submitted version.

## Funding

This research was supported by the earmarked fund for CARS (Project ID: CARS-20) and the National Natural Science Foundation of China (Project ID: 32060006).

## Conflict of interest

The authors declare that the research was conducted in the absence of any commercial or financial relationships that could be construed as a potential conflict of interest.

## Publisher’s note

All claims expressed in this article are solely those of the authors and do not necessarily represent those of their affiliated organizations, or those of the publisher, the editors and the reviewers. Any product that may be evaluated in this article, or claim that may be made by its manufacturer, is not guaranteed or endorsed by the publisher.

## Footnotes

^1^ http://www.indexfungorum.org/

^2^ http://www.mycobank.org/

^3^ http://mafft.cbrc.jp/alignment/server/

^4^ http://tree.bio.ed.ac.uk/software/figtree/

^5^ http://www.ncbi.nlm.nih.gov
